# Evaluation of the Suitability of Mammalian *In Vitro* Assays to Assess the Genotoxic Potential of Food Contact Materials

**DOI:** 10.3390/foods9020237

**Published:** 2020-02-22

**Authors:** Elisabeth Pinter, Bernhard Rainer, Thomas Czerny, Elisabeth Riegel, Benoît Schilter, Maricel Marin-Kuan, Manfred Tacker

**Affiliations:** 1 Department of Applied Life Sciences, University of Applied Sciences, FH Campus Wien, Helmut-Qualtinger-Gasse 2, 1030 Vienna, Austria; 2 Nestlé Research Center, Route du Jorat 57, 1000 Lausanne, Switzerland

**Keywords:** food contact material, genotoxicity, eukaryotic *in vitro* bioassays, safety assessment, non-intentionally added substances, packaging

## Abstract

Background: Non-targeted screening of food contact materials (FCM) for non-intentionally added substances (NIAS) reveals a great number of unknown and unidentified substances present at low concentrations. In the absence of toxicological data, the application of the threshold of toxicological concern (TTC) or of EU Regulation 10/2011 requires methods able to fulfill safety threshold criteria. In this review, mammalian *in vitro* genotoxicity assays are analyzed for their ability to detect DNA-damaging substances at limits of biological detection (LOBD) corresponding to the appropriate safety thresholds. Results: The ability of the assays to detect genotoxic effects varies greatly between substance classes. Especially for direct-acting mutagens, the assays lacked the ability to detect most DNA reactive substances below the threshold of 10 ppb, making them unsuitable to pick up potential genotoxicants present in FCM migrates. However, suitability for the detection of chromosomal damage or investigation of other modes of action makes them a complementary tool as part of a standard test battery aimed at giving additional information to ensure safety. Conclusion: improvements are necessary to comply with regulatory thresholds to consider mammalian genotoxicity *in vitro* assays to assess FCM safety.

## 1. Introduction

Food contact materials (FCMs) are complex mixtures made up of a wide variety of substances with different chemical and toxicological properties. Manufacturing of FCM involves the use of intentionally added substances (IAS) with functional and technical reasons in the manufacturing process or the final product. Some of these substances are regulated and their toxicological properties have been assessed. Moreover, unknown substances such as breakdown products, degradation products, reaction by-products and side reaction products of IAS, or other contaminants are also present, generating the so-called non-intentionally added substances (NIAS) [1]. These are present at low quantities and can be found in great numbers. NIAS are heterogeneous substances difficult to identify and, consequently, toxicological information is lacking [2,3]. Both IAS and NIAS might migrate into the packaged good and could potentially lead to an adverse health effect.

Assessing the safety of FCMs is consequently challenging as it intends to cover both the IAS and also the NIAS. Considering the lack of toxicological information to perform a risk assessment of FCM, the integration of a safe level concept into the approach is proposed. The European Food Safety Agency (EFSA) suggests applying the threshold of toxicological concern (TTC) concept for the assessment of FCM [4]. The TTC concept is a screening and prioritization approach for toxicological safety assessment, when there are limited toxicity data available or when hazard data are incomplete. Both criteria clearly correspond to the FCM situation, where structurally unknown substances are present [5,6]. The rationale of the TTC concept is based on the previous observation that for any chemical, there is a threshold of exposure below which no health concern is expected and there is no appreciable risk [7]. However, the application of this concept requires the exclusion of the following cohorts of concern: (1) the substances with genotoxic potential, with a threshold set at 0.15 µg person^−1^ per day; (2) The organophosphates or N-methyl carbamates responsible for neurotoxic effects, with a threshold set at 18 µg person^−1^ per day. Finally, (3) for substances with reactive functional groups, which structure suggests toxicity, Cramer Class III with a threshold set at 90 µg person^−1^ per day is applied [7,8,9]. Upon exclusion of genotoxic substances and organophosphate alerts, mixtures of substances can be handled as the Cramer Class III and a thorough analysis is not necessary unless they are present above a threshold of 90 µg person^−1^ per day [5,9]. Another approach includes the application proposed by European Regulation (EU) 10/2011 [10]. The regulation states an analytical limit of detection of 10 ppb corresponding to 0.01 mg·L^−1^ for substances, providing they are not carcinogenic, mutagenic, or reprotoxic. Non-authorized substances can be used behind a barrier layer, but it has to be proven that these do not migrate into the foodstuff. This approach has been widely used in the industry for the assessment of unknown substances due to the absence of more stringent regulations. This is why the approach is used as a pragmatic threshold for risk management purposes and has been proposed by [11] as a first step in the safety assessment of FCM mixtures. 

The aim of the present review is to assess the suitability and the performance of mammalian *in vitro* assays for the detection of genotoxic substances in FCM migrates and takes the above-proposed safe level approaches into consideration. The reason to apply *in vitro* assays to assess the safety of FCM is in alignment with the published guidelines on “Best Practices on the Risk Assessment of Non-Intentionally Added Substances (NIAS) in Food Contact Materials and Articles” by the International Life Science Institute (ILSI) Packaging Material task force [1] and a more comprehensive assessment on the use of bioassays for FCMs and their limits [11]. In the guideline by ILSI [1], the use of *in vitro* bioassays is recommended as a tool to estimate the risk of unknown substances migrating from an FCM. *In vitro* assays are recognized methods and have proven to successfully complement analytical methods in the characterization of unknown substances, as reported for potential endocrine effects [2,12,13,14].

However, focusing on genotoxicity assessment is challenging, as DNA-damage is a complex biological process involving several modes of actions (MoA), which determines the cellular fate and the severity of the hazard. The OECD (Organization for Economic Co-operation and Development) describes genotoxicity as a process that alters the structure, information content, or segregation of DNA that is not necessarily associated with mutagenicity [15]. Currently, a wide variety of bioassays are available to assess the genotoxic potential of chemicals and have been evaluated for their ability to correctly predict the adverse effects of pure substances and are often used as screening tools, e.g., during the development of new pharmaceuticals. Moreover, bioassays have already been applied for the genotoxicity assessment of complex mixtures, such as environmental samples, medical devices, impurities in pharmaceuticals and botanical preparations [16,17,18,19].

## 2. Genetic Toxicology 

Genetic toxicology focuses on substances that interact with nucleic acids and/or cause alterations to genetic material, with these effects taking place at levels where the substance is not cytotoxic [20]. Genotoxic substances can cause a wide spectrum of effects, which makes them very challenging to assess. These effects include interactions that reach from DNA mutations to strand breaks or complex DNA-damage signaling responses [21,22,23]. Genotoxicity tests often focus on a single MoA, hence a test battery is necessary to cover the various mechanisms of genotoxicity potentially occurring. 

In general, genotoxic effects can be categorized into direct or indirect effects. Direct-acting agents or their metabolites usually cause gene mutations, such as point mutations, insertions or deletions, or structural damage, including clastogenicity [24]. Due to the toxicological severity, these substances are treated in a non-threshold manner [4]. On the other hand, molecules/mixtures acting via an indirect MoA are those able to induce DNA-damage via other cellular mechanisms and are treated in a threshold manner. This is due to the fact that they are regarded as less potent compared to DNA reactive substances since a critical number of target sites must be occupied before a biological effect occurs [25]. Indirect DNA-damaging agents are, for example, aneugens that cause effects such as the inhibition of enzymes or proteins involved in the segregation of chromosomes during the cell cycle [26,27]. As aneugenic and clastogenic substances are not direct DNA-reactive mutagens [11], they are regulated with a Cramer Class III threshold [28,29,30].

*In vitro* genotoxicity tests can be classified according to the mechanism they are detecting. Direct-acting agents usually cause gene mutations and can be detected with assays such as the Ames test [31] or the mouse lymphoma assay (MLA) [32]. The MLA is a forward mutation assay where a mutation in the *hprt* or *tk* genes will lead to a deficiency of this protein, which makes the cells resistant to the cytotoxic effect of a selective medium [33]. Other effects caused by clastogenic or aneugenic substances do not lead to gene mutations but to strand breaks or disruption of the chromosomes so that other assays are used for the specific detection of these mechanisms. The chromosomal aberration (CA) [34], the sister chromatid exchange (SCE) [35], comet assay [36] or micronucleus assay (MN) [37] are commonly used tests for the detection of these effects. A newly developed method is the MN based on flow cytometric, which is thought to be an improved method compared to microscopic scoring and can give valuable additional information on the presence of clastogenic or aneugenic substances [38].

Both indirect- and direct-acting genotoxic substances may activate the cellular DNA-damage response. Some characteristic genes and proteins involved in the DNA-damage and repair signaling are the p53, GADD45α, γH2AX, and p21 [39]. These markers are activated upon DNA-damage, leading to a specific cellular response, such as apoptosis, senescence, cell cycle arrest, or DNA repair [40]. The importance of these markers in the development of cancer is widely acknowledged and they are promising points of intersection for genotoxicity assessment [41,42,43]. A number of reporter gene assays have been developed as fast screening methods of samples that can be conducted within a few days. Prominent examples are the p53 CALUX^®^ [44], the BlueScreen^TM^ HC [45], and the GreenScreen^TM^ HC [46], which were developed to precisely target these effects. Target gene activation can also be detected using high content screening, with microscopic or flow cytometry methods, focusing, for example, on the phosphorylation of the histone H2AX [43,47]. These assays are able to detect genes involved in DNA-damage and repair-signaling pathways, triggered when cells are exposed to a directly or indirectly acting genotoxic compound. However, the drawback of the DNA-damage response as a target for genotoxicity is the detection of non-genotoxicity related effects, which can also activate the same pathways, eventually caused by, e.g., cytotoxicity or oxidative stress, possibly resulting in a false-positive response. Another assay focusing on multiple endpoints is the ToxTracker^®^, which differs from most other tests as it is based on stem cells [48] and measures multiple pathways such as the ATR-Chk1, p53, and Nrf2. ToxTracker^®^ is a promising combination system which enables the assay to elucidate mechanistic effects and highlights the differences between true- and false-positives by a combination of these pathways. 

Depending on the recommended approach for genotoxicity testing, the assays are combined differently and can be part of a standard test battery or used as follow-up testing of equivocal or positive responses [21]. The comet assay, the MLA, and the MN are suggested for genotoxicity testing in combination with the Ames test [49] and will, therefore, be analyzed in more detail as part of this review. Assays focusing on the DNA-damage response will also be taken into account.

## 3. Mammalian *In Vitro* Assays for Genotoxicity Testing

*In vitro* assays can either be based on prokaryotic or eukaryotic cells and, depending on the mechanism of interest, different assays are suitable. Prokaryotic assays would exceed the scope of this review as the bacterial reverse mutation test (Ames) assay in the FCM context was recently evaluated [50]. For reasons of comparison, this assay will only be introduced for direct DNA reactive substances as it is recommended by the regulatory bodies for the testing of FCMs for mutagenic substances [11]. Eukaryotic cells have a complex metabolism and cell regulation systems and are thought to better reflect the human situation than the prokaryotic systems [51]. The most commonly used mammalian *in vitro* assays for genotoxicity testing are shown in Table 1. The following terminology was applied in the review: toxicological sensitivity describes the proportion of compounds testing positive in a given pool of genotoxic substances; the specificity, on the other hand, is the proportion of compounds tested as negative in a pool of given non-genotoxic substances [52,53]. Depending on the type and amount of substances tested, the toxicological sensitivity and specificity of an assay varies. 

Both the toxicological sensitivity and specificity are greatly affected by several parameters of a test system. Some influencing factors include, e.g., the cell line, metabolic activation system, cell viability and the incubation time. The selection of the cell line is critical to assess the DNA-damage potential, as specific metabolic pathways are required to trigger enzymatic reactions, generating metabolites with genotoxic potential. In case of a lack of metabolically active cell lines, exogenous activation of the metabolism (e.g., liver S9 fraction) is required. Depending on the S9 fraction and protocol used, different results might be obtained [54]. Also, various types of cells have a different tolerance towards S9 liver extract, affecting the result [55]. Particular relevance is given to potential cell viability adverse effects, as these can be a limiting factor upon the measurement of higher concentrations of genotoxic substances. Further, it might mask positive effects, leading to a false-negative response [56,57,58]. Hence, the simultaneous assessment of cytotoxicity is a crucial point for reliable results. Depending on the cell species and the tissue origin, different results are obtained, as HepG2 cells are less sensitive towards cytotoxic effects than, for example, HeLa or CHO cells [59].

**Table 1:** Overview of toxicological sensitivity and specificity of the most commonly used mammalian *in vitro* assays for the detection of genotoxicity. Values highlighted in green show a high (>75%), in orange a moderate (75% to 50%), or in red a low (<50%) toxicological sensitivity or specificity. The term toxicological sensitivity refers to the proportion of genotoxic substances correctly identified as positive in the assay. The specificity describes the substances correctly identified as negative in a pool of non-genotoxic substances [52,53].

Endpoint	Assay	Test System	Toxicological Sensitivity	Specificity	Number of Compounds Tested	Source
Gene Mutations	Ames Test OECD No. 471	Salmonella	59%	60%	541	[60]
			90%	87%	283	[61]
	SOS-Chromotest	Salmonella	38%	81%	177	[62]
	Rec-Assay	*Bacillus subtilis*	74%	62%	119	[63]
		*Escherichia coli*	76%	62%	277	[63]
	Mouse Lymphoma Assay (MLA) OECD No. 490	L5178Y	71%	44%	460	[63]
		L5178Y	73%	39%	350	[60]
Clastogenicity	Chromosomal Aberration (CA) OECD No. 473	CHL ^1^	69%	58%	255	[63]
		HPBL ^2^	51%	67%	123	[63]
	Sister Chromatid Exchange (SCE) OECD No. 479	CHL and CHO ^3^	68%	40%	438	[63]
		HPBL and HF ^4^	83%	35%	111	[63]
	Comet Assay OECD No. 489 (for *in vivo*)	HepaRG ^5^	44%	100%	16	[64]
		not indicated	88%	64%	95	[65]
Clastogenicity and Aneugenicity	Micronucleus (MN) OECD No. 487	HepaRG	73%	80%	16	[64]
		CHO-k1	80%	88%	62	[66]
		not given	79%	31%	115	[60]
		TK6 ^6^	88%	87%	48	[67]
		HPBL ^2^	79%	33%	38	[63]
DNA-Damage Response	p53 CALUX^®^	U2OS ^7^	82%	90%	60	[44]
	BlueScreen^TM^ HC	TK6	80%	100%	60	[45]
	GreenScreen^TM^ HC	TK6	90%	96%	43	[41]
		TK6	76%	88%	60	[44]
		TK6	67%	96%	71	[68]
	ToxTracker^®^	mES ^8^	85%	79%	27	[69]
		mES	95%	94%	54	[70]

^1^ CHL = Chinese hamster lung; ^2^ HPBL = human peripheral blood lymphocytes; ^3^ CHO = Chinese hamster ovary; ^4^ HF = human follicular lymphoma; ^5^ HepaRG = human hepatoma cell line; ^6^ TK6 = human lymphoblast thymidine kinase heterozygote; ^7^ U2OS = human bone osteosarcoma epithelial cell line; ^8^ mES = mouse embryonic stem cells.

Further, the duration of an assay is an important aspect, especially for high-throughput screening. The MLA, on the one hand, needs several weeks [32], while the reporter-gene assays detecting the activation of the DNA-damage response can be conducted in three to four days. 

The data presented in Table 1 shows some of the most important mammalian cell-based assays currently available to assess genotoxic potentials. The data were selected because of the amount of substances analyzed. The toxicological sensitivity and specificity were classified in high (>75%, green), moderate (75% to 50%, orange) or low (<50%, red) predictivity. According to those criteria, the assays detecting gene mutations, namely, MLA-*tk* and MLA-*hprt,* have moderate toxicological sensitivity and a low specificity [60,63]. Therefore, MLA-*tk* and MLA-*hprt* are considered as prone to false-positive results. For the assays detecting clastogenicity, both the SCE and the CA are of moderate to high toxicological sensitivity and low specificity [63] so they are also likely to give false-positive results. For the comet assay, conflicting data [64,65] was obtained depending on the substances analyzed. However, it shows to be promising and is a commonly used assay, which has already been used for several studies on the genotoxicity of FCMs and will be further assessed in this review. Depending on the cell line used, the MN showed high toxicological sensitivity especially for aneugenic substances [66] but seems to be prone to false-positive results, depending on the substance set [60]. Further, the used method had an effect as well, as more specific and sensitive results were obtained using a high-throughput flow cytometric MN assay [67]. Assays detecting DNA-damage responses prove to be of high predictivity, sensitivity, and specificity [41,44,45,70], making them suitable candidates for genotoxicity assessment, although only a limited amount of data is available and relatively few substances have been tested.

## 4. Detection of Low-Levels of Genotoxic Substances with Mammalian *In Vitro* Assays 

When assessing the presence of potential genotoxic substances in complex mixtures, such as FCM migrates, the question arises whether current mammalian cell-based assays are a suitable approach. To address this issue, an overview of the limits of biological detection (LOBD) of some *in vitro* genotoxicity assays introduced in the chapter ‘Mammalian *In Vitro* Assays for Genotoxicity Testing’ are listed. The limit of detection (LOD) in analytical chemistry reflects the lowest concentration where a substance can be reliably detected with a significant distinction from the blank. For bioassays, the concept of surrogated limits of biological detections was introduced [11]. This term is used to describe the ability of an assay to detect substances at certain concentration levels, with low calculated LOBDs (cLOBDs) corresponding to highly analytically sensitive assays. To calculate the LOBD, the lowest effect concentration (LEC) is used, which is the lowest concentration where a positive effect will be observed in a bioassay. In a literature survey, the cLOBDs of the most commonly used mammalian *in vitro* assays were evaluated and are presented in mg·L^−1^ for comparison of the respective tests and to determine the most analytically sensitive assays. It is important to point out that, here, the term ‘sensitivity’ differs from the term ‘toxicological sensitivity’ used in the previous sections as it does not refer to the ability of an assay to correctly detect true genotoxic substances but to reliably detect low quantities of a substance, and is from here on referred to as ‘analytical sensitivity’. Even though it is not expected that any of these substances migrating from an FCM will be found, this should provide an estimation of the assay’s ability to detect similar substances with genotoxic potential in an FCM migrate. To calculate the LOBDs, the collected data has to be normalized by taking the global concentration factor (GCF) into account. To better understand the assumptions of exposure considered, a brief description of the process applied to prepare extractable or migratable substances from FCMs is as follows. Migration is performed with a suitable solvent. Once migration simulation is completed, a concentration step (e.g., evaporation, solid phase extraction, liquid–liquid extraction, or lyophilisation) is performed. Finally, a solvent exchange takes place, where the migrated sample is transferred into a suitable solvent for bioassay application (e.g., dimethyl sulfoxide (DMSO)). Hence, the assumptions for the sample yield obtained are the following, which were also concluded by [50]

- a theoretical concentration factor of 1000 [71];
- the sample solvent is exchanged to 100% DMSO;
- no substances being lost during sample preparation or solvent exchange;
- a sample dilution factor of 100 (1% sample concentration in the cell culture medium);
- no cell viability artefacts being present, which might negatively affect the LOBD value.

With these assumptions, an overall factor of 10 is achieved for mammalian assays and 40 for the Ames test as it uses a dilution factor of 25 by applying 4% sample. To obtain the cLOBDs for the mammalian assays, the LECs found in the literature were divided by a factor of 10 to determine the theoretical detection in the FCM extract. 

The substances shown in Table 2 and Table 3 were chosen according to the recommended European Reference Laboratory for Alternatives to Animal Testing (EURL, ECVAM) workshop group list for the assessment of genotoxicity tests [72]. The substances were selected according to the amount of data available. In order to perform the FCM exposure correlation, the TTC exclusion criteria thresholds should be considered for (1) structural genotoxicity alert (0.15 µg/person per day; by assuming a consumption of 1 kg or 1 L of the food per day, this corresponds to 0.00015 mg·L^−1^ for direct and indirect DNA-damage); and (2) aneugenic substances, which are threshold dependent as Cramer Class III [26] with 90 µg·person^−1^ per day, corresponding to 0.09 mg·L^−1^. As a cLOBD of 0.00015 mg·L^−1^ is beyond reach with currently available bioassays, as concluded by [50], instead, a technical limit is used by applying European Regulation (EU) 10/2011. As already mentioned before, the EU proposes an analytical LOD of 10 ppb corresponding to 0.01 mg·L^−1^ [10]. Therefore, for the purpose of the present review, a threshold of 0.01 mg·L^−1^ was considered to assess direct DNA reactivity and an LOBD of 0.09 mg·L^−1^ for aneugens. 

Table 2 provides an overview of substances that cause gene mutations to determine the assay’s ability to detect this substance category. The MLA-*tk* is able to detect 11%, and the MLA-*hprt* 22% of the substances below the target LOBD of 0.01 mg·L^−1^. The Ames test is shown here for comparison, as it is the most commonly used assay for the detection of mutagenic substances. With this substance set, it is able to detect about 36% below the proposed threshold.

Table 3 provides an overview of the cLOBD values found for mammalian *in vitro* assays, which are able to detect clastogenic and aneugenic substances. The threshold for aneugenic substances corresponds to 0.09 mg·L^−1^, as shown before. The p53 CALUX^®^ was able to detect 29% of the substances at an appropriate level; however, no data could be found for seven substances. The BlueScreen^TM^ HC could detect 63% of the substances, making it the most analytically sensitive assay for this data set. Both for the MN and the comet, more data were available, and the MN could detect 63% and the comet 36% of the substances below the threshold of 0.09 mg·L^−1^. For the MN, the lowest cLOBDs were found when a flow cytometric high throughput method was used, but more data would be necessary for a full evaluation of the performance of this method. Although it is a promising assay, the ToxTracker^®^ is not included, as for this data set, little information on LEC values could be found and a comparison was concluded to be unreasonable.

## 5. Genotoxicity Testing of FCMs with Mammalian *In Vitro* Assays

An overview of studies assessing FCMs with mammalian *in vitro* assays is shown in Table 4. The studies were classified according to the genotoxicity assay performed and the FCM evaluated. Of the 11 studies listed, six used the comet assay, two the MN, and the BlueScreen^TM^ HC, the SCE and the CA were each applied once. The cell line most often used was HepG2 (5 out of 11 studies), which was applied for the MN and the comet assay. Except for the CA, which was performed with Chinese hamster lung cells (CHL), all assays were conducted with human cell lines. Out of the 11 studies analyzed here, two [102,103] applied exogenous metabolic activation while other bioassays were metabolically competent [104]. No information was available for the other studies. Cytotoxicity assessment, if performed, is indicated in the results column. 

Different FCM migration protocols were followed before bioassay application. As described in the previous section, if available, migration concentrations and the corresponding global concentration factors (GCFs) are shown in the table. Different solvents were used, such as 95% ethanol, water, Tenax, acetone or mineral water. Moreover, most studies also analyzed their samples with an additional analytical chemical method, such as GC–MS, LC–MS or HPLC. 

All this taken into consideration, a number of samples were positive for genotoxicity when using the comet assay for recycled paper [105] and polyethylene terephthalate (PET) [106]. In a study [105], several genotoxic substances were identified in a GC–MS analysis. These compounds ranged in concentration from 0.026 mg·kg^−1^ for benzophenone to 12 mg kg^−1^ for Michler’s ketone. Benzophenone was present in all positive samples. Other identified compounds were bisphenol A, 1,2-benzisothiazoline-3-one, 4-(dimethylamino)- benzophenone, 4,4’-bis(diethylamino)-benzophenone, pentachlorophenol, and 2,4,6-trichloroanisole. However, the samples tested as negative contained several of these substances in similar concentrations. Upon performance of a screening of the literature on *in vitro* genotoxicity testing with the substances found in the above-mentioned study [105], information could only be obtained for the Ames test with Michler’s ketone for TA98. For this, the cLOBDs were calculated using a factor of 40. With a cLOBD of 31 mg·L^−1^ for Michler’s ketone [77] and an actual concentration of 12 mg·kg^−1^, the Ames test would not have been able to detect this substance.

In a study [106], the positive results for genotoxic substances in mineral water stored in PET bottles were most likely caused by contamination in the distribution pipeline for the mineral water. This means that the PET bottles themselves are unlikely to have caused the positive signal in the bioassay. Considering all samples, the finding of a positive result was not only independent of the material but also of the cell line used for the assay so that genotoxic substances were found with HepG2, HL-60, and human leukocytes. 

Only negative results were scored by studies with the MN [107,108], the BlueScreen^TM^ HC [102], the CA [103], and the SCE [109]. The p53 CALUX^®^ was able to detect possible genotoxic effects in a study with samples such as a pizza box and paperboard with printing inks [110]. Only one of the six positive samples was further analyzed using a GC–QTOF, showing that the possible genotoxic substance, di-isobutyl phthalate, was present.

To sum up, the differences in migration conditions, food simulants, sample concentration methods and sample solvents made it difficult to compare the test results with each other. In addition, the different assays and cell lines used for the analysis further complicated the comparison.

**Table 4:** Overview of the studies conducted on the genotoxicity of food contact materials (FCM) with common mammalian *in vitro* assays. The studies are arranged according to the conducted assay.

Assay	Material	Simulant	Migration Protocol	Concentration Method	Sample Solvent	Result	Cell Type	+/-S9	Source
*BlueScreen^TM^ HC*	Paper	Tenax	10 d at 60 °C	-	Aqueous Water	0/3 positive Not cytotoxic No genotoxic substances in GC–MS and LC–MS analysis	TK6 ^1^	yes	[102]
*Chromosomal Aberration Test (CA)*	Polystyrene	Acetone	1 h at 40 °C, then addition of Methanol for 1 h at 40 °C	Evaporation GCF ^2^ = 1.5	Acetone	0/1 positive Not cytotoxic No genotoxic substances in GC–MS	CHL ^3^	yes	[103]
*Comet Assay*	Paper and Board	Water, 95% Ethanol, Tenax	Water: EN 645 (cold water extraction) and EN 647 (hot water extraction) 24 h at 20 or 80 °C 95% Ethanol: 24 h at RT ^4^ Tenax: 24 h at RT, 5 d at 50 °C, 10 d at 20 °C	Evaporation GCF = 10	95% Ethanol	0/20 positive Some samples cytotoxic No genotoxic substances in GC–MS	HepG2 ^5^	no ^6^	[104]
	Paper (recycled)	Ethanol	Refluxed for 2 h	Evaporation	DMSO ^7^	6/8 positive Cytotoxicity not evaluated Several genotoxic substances identified with GC–MS	HL-60 ^8^	no info	[105]
	Polyethylene-terephthalate (PET)	Mineral water	Storage for 1 to 12 months	Lyophilisation and evaporation	DMSO	6/12 samples positive for mineral water 5/12 samples positive for carbonated mineral water Cytotoxicity not evaluated No genotoxic substances in GC–MS	Human Leukocytes	no info	[106]
	PET	Mineral water	10 d at 40 °C and at RT	Solid Phase Extraction (SPE)	DMSO	some positive results, but not statistically significant Cytotoxicity not evaluated No genotoxic substances in GC–MS	Human Leukocytes	no info	[111]
	Polypropylene	-	Dissolving of the sample in ethyl acetate	Evaporation GCF = 0.1	DMSO	0/6 positive some cytotoxic effects No genotoxic substances in GC–MS and HPLC	HepG2	no info	[112]
*Micronucleus (MN)*	PET and glass	Water	10 d at 40, 50, 60 °C	Solid Phase Extraction GCF = 5	Ethyl acetate	0/4 sample pools positive Not cytotoxic No genotoxic substances in GC–MS	HepG2	no info	[107]
	PET and glass	Water	Exposure to sunlight for 2, 6, 10 d	Solid Phase Extraction GCF = 5	Ethyl acetate	0/4 sample pools positive Not cytotoxic Low concentrations of formaldehyde and acetaldehyde in GC–MS	HepG2	no info	[108]
*P53 CALUX^®^*	Paper and board	Ethanol	Refluxed for 4 h	Evaporation GCF = 1.3	Ethanol	6/20 positive Cytotoxicity not evaluated in one positive sample di-isobutyl phthalate identified with GC–QTOF	U2OS ^9^	no info	[110]
*Sister chromatid exchange (SCE)*	PET	Mineral water	8 weeks RT	-	Water	0/4 sample pools positive some cytotoxic effects No chemical analytical method conducted	Human Lymphocytes	no info	[109]

^1^ TK6 = human lymphoblast thymidine kinase heterozygote; ^2^ GCF = global concentration factor; ^3^ CHL = Chinese hamster lung; ^4^ RT = room temperature; ^5^ HepG2= human hepatocellular carcinoma cell line; ^6^ = HepG2 cells were used which proved to be metabolic competent so that the addition of a further metabolic system was not necessary [104]; ^7^ DMSO = dimethyl sulfoxide; ^8^ HL-60 = human leukemia cell line; ^9^ U2OS = human bone osteosarcoma epithelial cell line;.

## 6. Discussion

In the present review, the evaluation of the suitability of recognized mammalian cell methods to assess potential FCM genotoxicity is addressed. Conservative approaches, such as the TTC and EU regulations (10/2011), were proposed to manage the lack of toxicological data [1,10,11]. Within these opportunities and limitations, the suitability of detection methods to achieve the targeted thresholds defined by the TTC and EU 10/2011 were evaluated. Commonly used mammalian assays were assessed concerning their analytical and toxicological sensitivity and specificity to detect genotoxic substances and their suitability for the risk assessment of FCMs. As *in vitro* assays have the ability to estimate the effect of the overall mixture and possible sum effects, they are considered to be a helpful tool for the safety assessment of FCMs [1,11]. According to the published data, the mammalian assays discussed in this review were able to detect only 11% to 22% of the substances below the proposed technical threshold of 10 ppb [10] for direct-acting DNA reactive substances. This means that a genotoxic response at this level cannot be detected for a broad range of DNA reactive substances, possibly leading to the underestimation of a genotoxic risk. In contrast, a literature survey on the suitability of the Ames assay for FCM screening [50] concluded that a level of 10 ppb was feasible for about 50% of genotoxic standard substances out of a given pool of 16 genotoxins, making this assay currently better suited for the characterization of directly DNA-damaging substances in FCM migrates. For other substances, such as aneugens or those interfering with DNA synthesis, the mammalian assays could detect 29% to 63% at concentrations below the threshold of 0.09 mg·L^−1^, and therefore would be likely to detect them in FCM migrates as well.

Mammalian *in vitro* assays have been used as the method of choice for genotoxicity testing of FCMs by several authors. The comet assay was able to detect several possibly genotoxic responses in FCM migrates [105,106]. The authors included a chemical analysis in their study, which is important for identifying the substance responsible for the positive result. However, we found the specificity of some assays, such as the comet assay and the MN, to be of only moderate to low predictivity, as shown in Table 1, and could possibly lead to false-positive results. This raises the question whether some of the results indicating genotoxicity have to be considered as false-positives. 

In general, besides lacking specificity, another reason for false-positive results could be adverse cell viability effects. Therefore, additional testing for any toxic effect and the application of a toxicity threshold are essential. In the reviewed studies, most included a test for cytotoxicity, giving important background information on the samples and ruling out the possibility of false-positive results. Further, the application of the optimal cell line is essential for obtaining reliable and sensitive results. The report of an ECVAM workshop on avoiding false-positive results in *in vitro* genotoxicity testing assessed a variety of cell lines and found a large fluctuation within different cell lines and therefore recommends to stick to one, preferably human-based [57]. 

On the other hand, the possibility of obtaining false-negative results cannot be ruled out, which could be due to both low toxicological and analytical sensitivity. Most of these studies did not mention if a metabolic system was included in the test battery. This could also lead to false-negative results and therefore to the underestimation of a genotoxic effect. Moreover, none of the studies evaluated whether the LOBD of the assays was adequate to detect any genotoxic effect or was modulated by matrix effects in the presence of an FCM migrate. In addition, no data covering the impact of migration and sample preparation were discussed. Especially, the sample preparation procedure and selection of solvents deserve further attention when applied to volatile or degradable substances [113,114]. 

## 7. Conclusions

The application of biological assays to detect substances with genotoxic potential would enable the application of the TTC for FCM migrates to exclude cohorts of concern. However, the assays discussed in this review lack the analytical sensitivity to fully cover genotoxic effects at the proposed threshold of 10 ppb. When looking at DNA reactive substances, the bacterial mutation test demonstrated to be superior compared to its mammalian counterpart, the MLA. However, the analytical sensitivity of the former test still needs to be improved to meet TTC requirements. For indirect-acting genotoxic effects, the mammalian assays were able to detect only few of the substances at the proposed threshold and are therefore likely to detect them in FCM migrates as well. Yet, much data is missing so that a definite conclusion on their suitability is currently not possible. Also, the LOBDs obtained are only valid for pure substances and further research is necessary on whether this can be extrapolated to substances present in a complex migrate mixture. Nevertheless, mammalian assays have proven to be able to detect genotoxic substances in studies on FCM migrates for several different materials, even though the analytical limits for some compounds are not sufficient. However, a comparison of these results is not straight forward, as the procedures applied differ greatly. Therefore, a standardized protocol for FCM assessment would be necessary. Also, the possibility of false-positive or negative results cannot be ruled out. Further, controls with spiked genotoxic substances to simulate potential matrix effects and the assays’ ability to detect them are worth pursuing. 

Overall, according to the estimations presented in this review, the LOBD required to exclude genotoxic alerts in FCM cannot be achieved using currently available mammalian cell-based assays. However, through the improvement of the assays’ LOBDs by a factor of 10 to 100, most of the analyzed substances could be detectable below the threshold of 10 ppb. The use of mammalian assays for genotoxicity assessment of FCM migrates could lead to important additional information and could be used as a confirmation system to the Ames test if the LOBDs were greatly improved. 

## Figures and Tables

**Table 2:** Calculated limit of biological detection (cLOBD) for genotoxic substances that result in gene mutations as found in a literature survey for commonly used *in vitro* assays for detecting gene mutations with the global concentration factor (GCF) of 10 taken into account for mammalian assays and a GCF of 40 for the Ames test.

Mode of Action	Substance	MLA-*tk* (mg·L^−1^)	MLA-*hprt* (mg·L^−1^)	Ames (mg·L^−1^)
*Alkylating agents*	Cyclophosphamide	0.1 (+) [73]	0.1 (+) [74]	0.02 (+) [75]
	ENU (N-Ethyl nitrosourea)	-	0.04 * [76]	0.3 (-) [77]
	Methyl Methanosulphonate	0.6 [78]	0.6 * [79]	0.2 (-) [80]
*Polycyclic aromatic hydrocarbons*	Benzo-α-Pyrene	0.1 (+) [73]	0.01 (+) [81]	0.005 (+) [80]
	7,12-Dimethylbenzanthracene	0.05 (+) [73]	0.1 (+) [82]	0.2 (+) [83]
*Aromatic amines*	2-Acetylaminofluorene	4 (+) [84]	5 (+) [81]	0.003 (+) [80]
	2,4-Diaminotoluene	20 [85]	80 (+) [85]	0.2 (+) [86]
	Dimethyl Nitrosamine	1 (+) [78]	5 (+) [87]	0.2 (+) [88]
*Others*	Aflatoxin B1	0.001 (+) [84]	0.008 (+) [82]	0.00004 (+) [80]
	p-Chloroaniline—free base and HCl salt	19 (-) [73]	-	3 (+) [89]
	Cisplatin	-	0.03 (-) [90]	0.009 (-) [77]

(+): value obtained with S9 addition; (-): value obtained without S9; (+/-): value obtained both with and without S9; *: no information given whether an exogenous system was used; -: no data was found for a substance with the respective assay.

**Table 3:** Calculated limit of biological detection (cLOBD) for some aneugenic and clastogenic substances as found in a literature survey for the most commonly used mammalian *in vitro* assays, which cover these endpoints. A global concentration factor (GCF) of 10 was taken into account to normalize the data.

Mode of Action	Substance	p53 CALUX^®^ (mg·L^−1^) [44]	BlueScreen^TM^ HC (mg·L^−1^) [45]	Micronucleus (mg·L^−1^)	Comet (mg·L^−1^)
Probable aneugens and clastogens	4-Nitroquinoline Oxide	-	0.01 (-)	0.01 (-) [91]	0.001 (-) [92]
	Cadmium Chloride	Negative (+/-)	Negative (+/-)	0.0006 (-) [91]	4 (-) [93]
	Etoposide	0.6 (-)	0.01 (-)	0.002 (-) [94]	1 (-) [95]
	Hydroquinone	1 (-)	0.04 (-)	Negative (+/-) [66]	0.05 (-) [96]
	Taxol	0.03 (+/-)	0.003 (-)	0.008 (-) [91]	0.9 (-) [97]
	Azidothymidine	Negative(+/-)	Negative (+/-)	3 (+) [66]	50 (-) [98]
	5-Fluoruracil	-	-	-	0.07 (-) [99]
	Sodium Arsenite	0.001 (-)	0.06 (-)	0.01 (-) [66]	3 * [100]
	Methyl Nitrosurea	-	-	0.0008 (-) [91]	10 (-) [99]
	Doxorubicin	-	-	0.005 (-) [66]	0.07 (-) [101]
	Chloramphenicol	32+	Negative+/-	0.3 (+) [66]	-
	Bleomycin	-	-	0.5 (-) [53]	1 (-) [99]

(+): value obtained with S9 addition; (-): value obtained without S9; (+/-): value obtained both with and without S9; *: no information given whether an exogenous system was used; -: no data was found for a substance with the respective assay.
